# Health literacy and meeting breast and cervical cancer screening guidelines among Asians and whites in California

**DOI:** 10.1186/s40064-015-1225-y

**Published:** 2015-08-19

**Authors:** Tetine Sentell, Kathryn L. Braun, James Davis, Terry Davis

**Affiliations:** Office of Public Health Studies, University of Hawai‘i, 1960 East-West Road, Biomed, D-104, Honolulu, HI 96822 USA; ‘Imi Hale Native Hawaiian Cancer Network (U54CA153459), Papa Ola Lōkahi, 894 Queen Street, Honolulu, HI 96813 USA; Biostatistics Core, John A. Burns School of Medicine, Medical Education Building, Suite 401, 651 Ilalo Street, Honolulu, HI 96813 USA; Section of General Medicine, School of Medicine, Shreveport, Louisiana State University Health Sciences Center, 1501 Kings Highway, P.O. Box 33932, Shreveport, LA 71130-3932 USA

**Keywords:** Asian American, Cancer screening, Health literacy

## Abstract

**Objectives:**

Empirical evidence regarding cancer screening and health literacy is mixed. Cancer is the leading cause of death in Asian Americans, yet screening rates are notably low. Using a population-based sample, we determined if health literacy: (1) was associated with breast and cervical cancer screening, and (2) helped to explain Asian cancer screening disparities.

**Methods:**

We analyzed the 2007 California Health Interview Survey for Asian (Japanese, Chinese, Filipino, Korean, Vietnamese, other Asian) and white women within age groups relevant to US Preventive Services Task Force (USPSTF) screening guidelines: cervical: ages 21–65 (n = 15,210) and breast: ages 50–74 (n = 11,163). Multilevel logistic regression models predicted meeting USPSTF screening guidelines both with and without self-reported health literacy controlling for individual-level and contextual-level factors.

**Results:**

Low health literacy significantly (p < 0.05) predicted lower cancer screening in final models for both cancer types. In unadjusted models, Asians were significantly less likely than whites to receive both screening types and significantly more likely to report low health literacy. However, in multivariable models, the addition of the low health literacy variable did not diminish Asian vs. white cancer screening disparities.

**Conclusions:**

Self-reported health literacy predicted cervical and breast cancer screening, but was not able to explain Asian cancer screening disparities. We provide new evidence to support a relationship between health literacy and cancer screening. Health literacy is likely a useful focus for interventions to improve cancer screening and ultimately reduce the burden of cancer. To specifically reduce Asian cancer disparities, additional areas of focus should be considered.

## Background

Adult health literacy is an established correlate to many cancer-related predictors and outcomes, associated with more advanced cancer at detection and other factors that may contribute to diminished knowledge, access, and desire for cancer screening, including poorer understanding of cancer communication (Davis et al. 2002; Scott et al. 2002; Berkman et al. 2011; Bennett et al. 1998). However, current evidence regarding the relationship between cancer screening and health literacy is mixed (Oldach and Katz 2014). While a recent literature review found a trend towards a relationship between low health literacy and less cancer screening, the authors noted significant methodological limitations in existing research, including outcomes that did not follow recommended cancer screening guidelines and lack of adjustment for key confounding factors (Oldach and Katz 2014).

Contextual variables are one group of confounding factors missing from most studies of health literacy and cancer screening. Contextual factors, such as community-level ethnic density and the supply of screening facilities, have been associated with cancer screening beyond individual-level factors (Datta et al. 2006; Mobley et al. 2010; Pourat et al. 2010; Kandula et al. 2009). Considering these factors in research on cancer screening generally is important. They may be particularly important in studies of health literacy and cancer screening. For instance, individuals with lower health literacy in communities with a large supply of screening facilities may face fewer logistic challenges in understanding and acting on cancer screening-related health information (e.g., relevant bus schedules, hours of operation) than individuals with lower health literacy in communities without few or no screening facilities.

One population for whom this issue is particularly important is Asian Americans. Cancer is the leading cause of death among Asian Americans, yet cancer screening rates remain lower for Asian Americans than for many other racial/ethnic groups in the United States (U.S.) (Liss and Baker 2014; Gomez et al. 2007). Socioeconomic status, linguistic factors, and availability of and access to care issues that underlie many health disparities have been, at best, only partly able to explain Asian American cancer screening disparities (Liss and Baker 2014; Jerant et al. 2008). Research is urgently needed to understand the low rates of cancer screening among Asian Americans, particularly considering communication-related factors and Asian subgroups (Liss and Baker 2014).

Health literacy can be measured in a variety of ways (Baker 2006). While health literacy is conceived of, and increasing being quantified, as a multifaceted concept, health literacy has traditionally been measured by assessing reading ability and/or numeracy skills (either objectively measured or by self-report). More recent health literacy assessment tools have included other skills such as interpreting maps and understanding verbal information (Haun et al. 2014). Health literacy may help to explain the low rates of cancer screening among Asian Americans. A recent study found that more Asian Americans self-reported low health literacy than whites and that self-reported low health literacy was associated with low colorectal cancer screening in Asian Americans (Sentell et al. 2013). Studies have illuminated significant health literacy challenges in some Asian American communities (Todd and Hoffman-Goetz 2011; McWhirter et al. 2011; Simon et al. 2014; Leung et al. 2014; Sentell et al. 2015). Additionally, previous studies of white, African American, and Latino populations have found self-reported literacy and health literacy to be stronger explanatory factors for some health disparities than race/ethnicity (Sentell and Halpin 2006; Bennett et al. 2009; Howard et al. 2006). Together, these findings strongly suggest that low healthy literacy may be a critical factor to explain low rates of Asian American cancer screening.

Health literacy may provide insights into cancer screening differences between Asian American subgroups as well. The Asian American label encompasses at least 50 ethnic groups with distinct cultures and languages. Health literacy is known to vary across Asian American subgroups (Sentell and Braun 2012) as does cancer incidence, mortality, and risk factors (McCracken et al. 2007; Miller et al. 1996, 2007; Gomez et al. 2010). For instance: cervical cancer incidence in Vietnamese women is five times higher than in white women and seven times higher than in Japanese women (Miller et al. 1996). Distinct Asian American populations must be disaggregated to identify unique health risks, disparities, and interventions (McCracken et al. 2007; Miller et al. 2007; Gomez et al. 2010).

Our first study goal was to determine if health literacy was associated with breast and cervical cancer screening after considering individual and contextual-level confounders. We used a population-based sample with recommended cancer screening guidelines as study outcomes. Breast and cervical cancer were considered as they represent significant public health problems, have strong methods for early detection, have excellent evidence that screening is useful, and show disparities for whites compared to Asian Americans (U.S. Department of Health and Human Services 2000; Liss and Baker 2014; Gomez et al. 2007). Our hypothesis was that low health literacy would be associated with lower breast and cervical cancer screening even after considering key confounding factors.

If health literacy was associated with cancer screening overall, our second study goal was to determine if this factor helped to explain Asian American cancer screening disparities. We considered this issue overall for (1) Asian Americans vs whites generally, and (2) for Asian American subgroups compared to whites. Our hypothesis was that low health literacy would help to explain Asian American cancer screening disparities compared to whites for both breast and cervical cancer.

## Methods

### Study design

We analyzed the 2007 California Health Interview Survey for Asian (Japanese, Chinese, Filipino, Korean, Vietnamese, other Asian) and white women within age groups relevant to US Preventive Services Task Force (USPSTF) 2009 breast cancer screening recommendations and 2003 cervical cancer screening recommendations (while screening recommendations for breast and cervical cancer have changed over time, the recommended ages for, and frequencies of, screening mammography and Pap smears have not changed since 2009 and 2003, respectively). Multilevel logistic regression models predicted meeting USPSTF screening guidelines both with and without self-reported health literacy controlling for individual-level and contextual-level factors.

### Data

The 2007 California Health Interview Survey (CHIS) was used for analyses. The CHIS is a random-digit-dial telephone survey administered by the UCLA Center for Health Policy Research (CHIS 2015a). As California is home to approximately one of four Asians in the U.S., the population-based CHIS included substantial sample sizes of Asian Americans (U.S. Census Bureau 2012). CHIS interviews are available in Asian languages (Mandarin, Cantonese, Korean, and Vietnamese), allowing for participation by those with limited English proficiency (LEP) (CHIS 2007).

### Screening outcome variables

#### Breast cancer

Women 30 and older were asked if they ever had a mammogram. If yes, they were asked, “How long ago did you have your most recent mammogram?” Following U.S. Preventive Services Task Force (USPSTF) guidelines, we defined meeting breast cancer screening guidelines as having had a mammogram in the past 1–2 years for women *aged 50*–*74* (USPSTF 2009).

#### Cervical cancer

Women 18 and older who were not currently pregnant and without a hysterectomy were asked if they ever had a Pap smear. If yes, they were asked “How long ago did you have your most recent Pap smear test?” Following the USPSTF guidelines, meeting cervical cancer screening guidelines was defined as a Pap smear in the past 1–3 years for women aged 21–65 (USPSTF 2003).

### Independent variables

#### Health literacy

Based on previous research, health literacy was assessed with two questions: (1) “When you get written information at a doctor’s office, would you say that it is very easy, somewhat easy, somewhat difficult, or very difficult to understand?” and (2) “When you read the instructions on a prescription bottle, would you say that it is very easy, somewhat easy, somewhat difficult, or very difficult to understand?” (Sentell et al. 2013; Health Research for Action 2009). Respondents could report not getting written information (<4 % of all respondents) or not using prescription medicine (<2 % of all respondents). In the full sample, <1 % of the sample lacked a response to either question. Low health literacy was defined as responding that (1) written information at the doctor’s office is “somewhat” or “very difficult” to understand and/or (2) instructions on a prescription bottle are “somewhat difficult” or “very difficult” to understand.

#### Race/ethnicity

Race/ethnicity was self-reported and defined as Asian (Chinese, Filipino, Japanese, Korean, Vietnamese, and other Asian American) or non-Hispanic white.

#### Individual-level controls

Final models included Limited English Proficiency (LEP), defined as self-reporting speaking English “not well” and “not at all” (among those who spoke another language at home besides English) (Cordasco et al. 2011) as well as age (continuous 18–85), education (less than high school, high school graduate, college graduate, more than college), poverty (≤100 % of poverty level vs. not), living in a rural area (vs. not), current insurance (vs. none), born in the U.S. (vs. elsewhere), and marital status (married vs. other).

#### Contextual-level controls

Contextual factors were included based on evidence that community-level factors predict health care access, health behaviors, and cancer screening generally and in Asian Americans specifically (Datta et al. 2006; Mobley et al. 2010; Pourat et al. 2010; Kandula et al. 2009). To create these variables, CHIS data was linked to contextual data compiled by RTI International and named the RTI Spatial Impact Data (RTI Spatial Impact Factor 2012). Contextual information was obtained at the level of the Medical Service Study Area (MSSA). MSSA area designations are designed to better capture socio-economic and geographical disparities in California than county borders (Office of Statewide Health Planning and Development 2003). MSSA-level data was used from the 2000 Census to note the external environment and from Centers for Medicaid and Medicare to include relevant health system access factors. Specific contextual variables were Asian ethnic density, measured by the proportion of non-Hispanic Asian Americans in the MSSA, contextual poverty, measured by the percent of people 65 years and older who lived in poverty within the MSSA, and the average distance to provider in the MSSA specific to each cancer type (i.e., to the closest mammography provider from 2006 and the number of clinically active OB-GYNs in the MSSA in 2000–2001). These were linked to CHIS data using respondents’ census tract information available through the CHIS Data Access Center (CHIS 2015b).

#### Statistical methods

After exclusions, unweighted samples were: cervical: 15,210 and breast: 11,163. Numbers were slightly (<1 %) smaller in regression models because of missing data on covariates. Data were analyzed in SAS 9.3 (2011; Cary, NC, USA: SAS Institute, Inc) using appropriate methods to correct for the complex sample design. Study aims were tested with multi-level logistic regressions (Gelman and Hill 2007). Relationships for unadjusted low health literacy were considered for each type of cancer screening. Separate multilevel logistic regressions were performed for achieving each of the two cancer screening guidelines for (1) Asian Americans vs whites, and (2) Asian American subgroups compared to whites. In both cases, the model was run first without the health literacy variable (Model A) and then with the health literacy variable included (Model B).

The significance of the health literacy variable in Model B tested study aim 1 (Is health literacy associated with cancer screening?). To test study aim 2 (Can health literacy explain Asian cancer screening disparities?), we followed previous research and used changes in size and statistical power of the Asian ethnicity variables across the two models to consider the explanatory power of the health literacy variable in explaining Asian American cancer screening disparities (Sentell and Halpin 2006). This study was deemed exempt by the University of Hawai‘i Institutional Review Board under federal exemption 4.

## Results

Table 1 provides descriptive information about each of the two cancer screening samples. Overall, 85.2 % met breast cancer screening guidelines and 87.2 % met cervical cancer screening guidelines. Percentages varied significantly for Asian Americans vs. whites, with Asians having significantly (p < 0.05) lower screening rates than whites in both samples. Eleven percent of the breast cancer sample and 12.2 % of the cervical cancer sample reported low health literacy. In both samples, low health literacy varied significantly (<0.0001) for Asian Americans vs. whites, with Asians having higher rates of low health literacy. Significant variation was seen for all demographic variables for Asian Americans vs. whites in both samples.

**Table 1:** Study Demographics from 2007 California Health Interview Survey (CHIS)

	Mammography sample				Pap sample			
	Women 50–74				Women 21–65			
	All	Asian	White		All	Asian	White	
Unweighted N	11,163	957	10,206		15,210	1950	13,260	
		%	%	P value		%	%	P value
Received screening	85.2	82.0	85.9	*0.022*	87.2	79.3	89.3	*<0.0001*
Low health literacy	11.4	19.3	9.8	*<0.0001*	12.2	17.3	10.7	*<0.0001*
Demographics								
LEP	6.1	1.0	32.6	*<0.001*	4.63	1.0	19.3	*<0.0001*
Education				*<0.0001*				*<0.0001*
<HS	7.3	14.2	6.0		5.0	7.0	4.5	
HS/some college	52.1	38.9	54.8		46.7	34.4	50.1	
College	25.3	35.2	23.4		30.7	39.7	28.2	
Grad school	15.2	11.7	15.9		18.9	17.2	17.1	
Age group				*<0.0001*				<0.0001
21–49	–	–	–		63.6	73.8	60.8	
50–64	74.9	69.2	76.1		34.9	25.0	37.6	
65+	25.1	30.8	23.9		1.5	1.3	1.5	
Below/near poverty	6.64	15.7	4.8	*<0.0001*	6.9	10.6	5.9	*<0.0001*
Rural	15.0	3.4	17.4	*<0.0001*	13.5	4.8	15.9	*<0.0001*
Insured		87.1	92.9	*<0.0001*	85.9	80.7	87.3	*<0.0001*
Married	67.0	76.9	65.0	*<0.0001*	63.8	67.8	69.2	*0.02*

All percentages are weighted

P values of less than 0.05 were italicized

Table 2 shows the characteristics by cancer screening by low health literacy for Asian Americans vs. whites overall and for Asian American subgroups specifically. For both Asians and whites, significant differences were seen for cancer screening by low health literacy. For instance, only 66.9 % of Asian Americans with low health literacy received pap screening that met guidelines, compared to 79.3 % of those without low health literacy (p < 0.001). Similar trends were seen for most Asian American subgroups; individuals with low health literacy had lower cancer screening rates compared to those without low health literacy.

**Table 2:** Unadjusted Percentages Cancer Screening by Low Health Literacy (LHL) in 2007 California Health Interview Survey

	% Mammography				% Pap			
	All	LHL	Not LHL	P value	All	LHL	Not LHL	P value
All	85.2	78.4	85.2	*<0.0001*	87.2	83.96	88.16	*0.0002*
Race/ethnicity								
White	85.9	78.4	86.7	*0.0024*	89.3	85.7	89.8	*0.002*
Asian Overall	82.0	72.3	84.3	*0.005*	79.3	66.9	81.9	*0.0001*
Asian Subgroup								
Chinese	85.5	73.2	92.4	*<0.0001*	77.8	71.5	80.5	0.139
Japanese	87.3	97.6	86.2	0.064	80.0	77.0	80.5	0.745
Filipino	83.0	76.2	83.2	0.64	79.5	59.7	80.9	0.061
Korean	64.3	66.3	63.5	0.82	77.0	63.1	81.1	*0.014*
Vietnamese	80.0	64.1	83.1	0.21	83.8	69.1	86.1	*0.06*
Other Asian	81.4	66.4	86.0	0.217	79.2	54.4	83.4	*0.0025*

Table 3 shows the results of multivariable models predicting both types of cancer screening for Asian Americans vs. whites first in the model excluding low health literacy (Model A) and then in the model including low health literacy (Model B).

**Table 3:** Multivariable logistic regression models (Model A: Without low health literacy; Model B: With low health literacy) for Asians overall vs. White predicting receiving recommended screening in the 2007 California Health Interview Survey (CHIS)

	Received Mammography		Received Pap	
	Unweighted n = 11,163		Unweighted n = 15,210	
	Model		Model	
	A	B	A	B
	OR (95 % CI)	OR (95 % CI)	OR (95 % CI)	OR (95 % CI)
Low health literacy	–	0.72 (0.57–0.90)	–	0.71 (0.60–0.83)
Race/ethnicity				
White	Reference	Reference	Reference	Reference
Asian	0.75 (0.57–0.97)	0.75 (0.58–0.98)	0.36 (0.31–0.42)	0.36 (0.31–0.42)
Demographics				
LEP	0.83 (0.58–1.20)	0.89 (0.62–1.30)	0.77 (0.60–0.99)	0.83 (0.64–1.07)
Education				
<HS	0.52 (0.36–0.73)	0.54 (0.38–0.77)	0.49 (0.37–0.66)	0.52 (0.39–0.69)
HS/Some college	0.79 (0.61–1.01)	0.79 (0.61–1.02)	0.57 (0.47–0.69)	0.58 (0.48–0.70)
College	0.87 (0.66–1.15)	0.87 (0.66–1.15)	0.84 (0.69–1.03)	0.84 (0.69–1.03)
Graduate degree	Reference	Reference	Reference	Reference
Age	1.04 (0.93–1.18)	1.04 (0.92–1.17)	0.79 (0.75–0.83)	0.78 (0.75–0.82)
Below/near poverty	0.69 (0.52–0.92)	0.72 (0.54–0.95)	0.68 (0.56–0.82)	0.70 (0.58–0.85)
Rural	0.92 (0.69–1.22)	0.91 (0.68–1.21)	0.80 (0.65–0.99)	0.80 (0.64–0.98)
Insured	5.07 (4.04–6.35)	5.01 (4.00–6.28)	2.85 (2.47–3.28)	2.84 (2.47–3.28)
Married	1.56 (1.32–1.85)	1.56 (1.32–1.84)	1.92 (1.70–2.18)	1.92 (1.70–2.18)
Context variables				
Distance/supply	0.99 (0.97–1.01)	0.99 (0.97–1.01)	1.00 (0.99–1.01)	1.00 (0.99–1.01)
% Elder poverty	0.38 (0.02–7.06)	0.38 (0.02–6.88)	0.50 (0.04–5.70)	0.53 (0.05–6.03)
% Community Asian density	3.64 (1.33–9.98)	3.66 (1.34–10.02)	4.23 (1.71–10.47)	4.32 (1.75–10.67)

As in the test for study aim 1, health literacy was significant in both screening models. Low health literacy was consistently associated with lower breast and cervical cancer screening. The test of study aim 2 was also consistent. The addition of the low health literacy, while significantly associated with cancer screening, did not diminish the Asian vs. white screening disparities.

Specifically for the breast cancer screening model, the Asian American vs. white variable was statistically significant in Model A. Low health literacy was significant in Model B, but the inclusion of this variable did not impact the statistical significance or strength of the association of any of the variables in the model, including the Asian American vs. white variable. Similarly, in cervical cancer screening, the Asian American vs. white variable was statistically significant in Model A and low health literacy was significant in Model B, but the addition of this variable did not impact the statistical significance or strength of the association of any of the variables in the model, including the Asian vs. white variable with one exception. LEP was no longer associated with cervical cancer screening in the model after including low health literacy.

Other significant factors associated with breast cancer screening were education, marital status, and insurance, which had a particularly strong relationship with screening. Community Asian American density was associated with higher odds of breast cancer screening. Distance to mammography screening facilities was also marginally associated with lower odds of breast cancer screening. Other significant factors associated with cervical cancer screening were age, education, marital status, English proficiency, rural (vs. urban) residence, and insurance. Community Asian American density was associated with higher odds of cervical cancer screening. Supply of OB/GYNs was also marginally associated with lower odds of cervical cancer screening.

Table 4 shows the odds ratios for both types of cancer screening for Asian American subgroups vs. whites for Models A and B. Results were similar to those in the Asian American vs. white comparisons; in all cases, low health literacy was significant in Model B, but did not impact the statistical significance or strength of the association of the Asian Americans subgroup variables that were significant in Model A.

**Table 4:** Multivariable logistic regression models predicting cancer screening (Model A: Without low health literacy; Model B: With low health literacy) for Asian American subgroups (compared to Whites) in the 2007 California Health Interview Survey (CHIS)

	Received Mammography		Received Pap	
	Unweighted n = 11,163		Unweighted n = 15,210	
	Model		Model	
	A	B	A	B
	OR (95 % CI)	OR (95 % CI)	OR (95 % CI)	OR (95 % CI)
Low health literacy	–	0.70 (0.56–0.89)	–	0.73 (0.62–0.86)
White	Reference	Reference	Reference	Reference
Japanese	0.95 (0.49–1.84)	0.95 (0.49–1.83)	0.32 (0.21–0.50)	0.33 (0.21–0.51)
Chinese	1.03 (0.65–1.63)	1.10 (0.69–1.76)	0.30 (0.23–0.38)	0.31 (0.24–0.40)
Filipino	0.59 (0.41–0.85)	0.58 (0.40–0.83)	0.34 (0.27–0.43)	0.34 (0.27–0.42)
Korean	0.58 (0.31–1.07)	0.58 (0.31–1.07)	0.46 (0.32–0.67)	0.46 (0.32–0.67)
Vietnamese	1.36 (0.69–2.68)	1.29 (0.65–2.54)	0.69 (0.47–1.02)	0.66 (0.45–0.98)
Other Asian	0.65 (0.34–1.25)	0.69 (0.36–1.34)	0.35 (0.26–0.47)	0.36 (0.27–0.48)

## Discussion

Low health literacy as measured by self-reported understanding of print health-related materials was significantly associated with lower breast and cervical cancer screening when other individual and contextual-level factors were controlled. This study provides new, strong evidence from a population-based sample that low health literacy is associated with these two recommended cancer screening guidelines even after controlling for individual and contextual-level confounders. This association highlights the potential of health literacy as a practical focus for interventions designed to improve cancer screening and to ultimately reduce the burden of cancer.

While Asian Americans had significantly higher rates of self-reported low health literacy compared to whites, the addition of low health literacy to the multivariable model did not explain Asian American cancer screening disparities generally or for Asian subgroups compared to whites. Asian American variables were significant both before and after the addition of low health literacy to the model, indicating that additional factors are needed to account for Asian American vs. white screening disparities. Screening rates for the two types of cancer screening were high overall (>80 %), so those who did not receive screening may be a particularly marginalized group. As has been found in previous research (Liss and Baker 2014; Jerant et al. 2008), our study indicates that those at risk among Asian American populations may not always be readily identified through traditional SES, access, language, or health communication measures. We had hypothesized that health literacy would provide new insight into these disparities, but did not find this to be the case. However, we measured health literacy through a subjective assessment across one health literacy dimension. It is possible that more comprehensive measures of health literacy may be a fruitful area for future research to help explain Asian cancer screening disparities (Han et al. 2014; Haun et al. 2014).

Our findings should be useful to research, practice, and policy. Overall, results suggest that health literacy focused interventions, especially those aligned with the ways in which this study measured health literacy (as comfort with reading print materials), may improve screening rates across multiple cancer screening types. Examples might be assistance with reading print material or strategies that eliminate the need for, or simplify the burden of, reading written materials. As Asian Americans have lower levels of health literacy than whites, health literacy-focused interventions could improve screening rates in Asian American populations. Still, additional areas of focus may be needed to reduce Asian American vs. white cancer screening disparities, including intensive cultural tailoring of materials and system navigation assistance (Carney et al. 2014; Wang et al. 2010; Heo and Braun 2014).

Notably, the factor associated most strongly with screening across both models was health insurance. If outreach to Asian Americans around the Affordable Care Act (ACA) is successful, the ACA expansion may be important to improving cancer screening rates and potentially reducing screening disparities for Asian Americans (Liss and Baker 2014). Reaching Asian American communities may be a challenge, however, due to linguistic diversity and uncertainty regarding eligibility (Rao 2013). In related findings, Asian ethnic density was associated with higher breast and cervical cancer screening. Previous research has found density of Asian Americans in a community to impact Asian Americans’ health behaviors (Kandula et al. 2009). Given our findings of higher screening prevalence in communities with high density of residents of Asian heritage, interventions may thus be especially needed in areas with low Asian densities. The ability to understand and act on cancer screening-related health information may vary with the general health knowledge and resources available within a sub-community. Distance to a screening and the relevant supply of providers were also marginally associated with all three outcomes, supporting previous research that such contextual information is important to screening (Datta et al. 2006; Mobley et al. 2010; Pourat et al. 2010; Kandula et al. 2009). These may also be important targets of policy, particularly as there are concerns about supply with the greater access to insurance under the ACA (Hill 2012).

This study has a number of strengths. We considered not just individual-level data, but also contextual-level data, using the CHIS data set, which has provided core knowledge around individual correlates for Asian American and cancer screening disparities (Gomez et al. 2007) as well as contextual correlates linked to cancer screening generally (Pourat et al. 2010). The CHIS allows for a substantial sample of Asian Americans, a non-English based measure of health literacy, and strong control variables from a data set for which the data integrity is carefully monitored.

This study also has some limitations. It focuses on one state, which may not represent Asian American populations outside California. Yet the California-specific context of this data is also an asset, allowing comparisons across areas with a range of varying Asian American ethnic densities and linguistic characteristics. These trends are expected to become increasing relevant within other US states and communities, and should already be of interest for other communities with substantial Asian communities who still lack detailed Asian population-level data.

Another limitation was that our mammography question did not confirm whether it was a screening or diagnostic mammogram. Usually, a diagnostic mammogram follows a screening mammogram, i.e., about 15 % of women who receive a screening mammogram are called by for additional testing. It also may be true that some women, particularly those with limited health literacy may not undergo a mammogram until a lump is detected, and thus her first mammogram may be diagnostic.

The 2007 CHIS lacks some variables (e.g., health beliefs and previous cancer history, avoidance of the health care system) associated with cancer screening (Chen 2005). While the 2007 CHIS health literacy measures have been used previously (Sentell et al. 2013; Sentell and Braun 2012; The Commonwealth Fund  2007; Health Research for Action 2009) and are similar to validated self-report measures (Chew et al. 2008), they are self-reported measures around one health literacy domain and have not been validated compared to other health literacy instruments. Other types of health literacy may be more relevant for Asian American cancer screening (Baker 2006). Furthermore, while previous studies have found self-reported literacy and health literacy to be stronger explanatory factors for some health disparities than race/ethnicity (Sentell and Halpin 2006; Bennett et al. 2009; Howard et al. 2006), this relationship is not consistent across all outcomes, suggesting that literacy and health literacy may work distinctly in different health areas (Bennett et al. 2009; Fransen et al. 2014). Also, the CHIS only included a health literacy measures in the 2007 data. It would be useful to consider more recent data. Including health literacy measures in large, population-based surveys such as the CHIS would support such research.

## Conclusions

This study finds that low health literacy is an important factor associated with cervical and breast cancer screening in a large, population-based sample. General health literacy interventions may improve cancer screening rates in the population overall across multiple cancer types. However, differences in low health literacy do not appear to explain Asian American vs. white cancer screening disparities specifically. Interventions focused on reducing Asian American screening disparities specifically may need to include additional strategies, such as pathways to insurance or culturally-focused interventions.
